# Vomiting in Pregnancy

**Published:** 1891-01

**Authors:** 

**Affiliations:** Philadelphia


### VOMITING IN PREGNANCY.

BY PROF. GOODELL, PHILADELPHIA.

| | | |
|---|---|---|
| R Cerii oxalat | . . . . . . . . | gr. j. |
| Ipecacuanhae | . . . . . . . . | gr. j. |
| Creosoti | . . . . . . . . | gtt. ij. |

M. Sig.—This is to be taken every hour until nausea is controlled.

